# Pumpkin (*Cucurbita moschata*) HSP20 Gene Family Identification and Expression Under Heat Stress

**DOI:** 10.3389/fgene.2021.753953

**Published:** 2021-10-14

**Authors:** Yanping Hu, Tingting Zhang, Ying Liu, Yuxin Li, Min Wang, Baibi Zhu, Daolong Liao, Tianhai Yun, Wenfeng Huang, Wen Zhang, Yang Zhou

**Affiliations:** ^1^ Key Laboratory of Vegetable Biology of Hainan Province, Hainan Vegetable Breeding Engineering Technology Research Center, The Institute of Vegetables, Hainan Academy of Agricultural Sciences, Haikou, China; ^2^ Key Laboratory for Quality Regulation of Tropical Horticultural Crops of Hainan Province, School of Horticulture, Hainan University, Haikou, China

**Keywords:** *Cucurbita moschata*, heat shock protein 20, gene family, gene expression, heat stress

## Abstract

Pumpkin (*Cucurbita moschata*) is an important cucurbit vegetable crop that has strong resistance to abiotic stress. While heat shock protein 20 (HSP20) has been implicated in vegetable response to heat stress, little is known regarding activity of HSP20 family proteins in *C. moschata*. Here, we performed a comprehensive genome-wide analysis to identify and characterize the functional dynamics of the *Cucurbita moschata HSP20* (*CmoHSP20*) gene family. A total of 33 *HSP20* genes distributed across 13 chromosomes were identified from the pumpkin genome. Our phylogenetic analysis determined that the CmoHSP20 proteins fell into nine distinct subfamilies, a division supported by the conserved motif composition and gene structure analyses. Segmental duplication events were shown to play a key role in expansion of the *CmoHSP20* gene family. Synteny analysis revealed that 19 and 18 *CmoHSP20* genes were collinear with those in the cucumber and melon genomes, respectively. Furthermore, the expression levels of pumpkin *HSP20* genes were differentially induced by heat stress. The transcript level of *CmoHSP20-16*, *24* and *25* were down-regulated by heat stress, while *CmoHSP20-7*, *13*, *18*, *22*, *26* and *32* were up-regulated by heat stress, which could be used as heat tolerance candidate genes. Overall, these findings contribute to our understanding of vegetable HSP20 family genes and provide valuable information that can be used to breed heat stress resistance in cucurbit vegetable crops.

## Introduction

Plants are typically afflicted by a wide variety of abiotic and biotic stresses during growth, including as extreme heat, cold, drought, elevated salinity levels, and pest and pathogen infestations. Over evolutionary time, plants have evolved a set of unique defenses including different morphological, molecular and physiological mechanisms or adaptations to avoid exposure to adverse effects (Wang et al., 2004). Heat shock protein (HSP) are often associated with plant responses to temperature stress, heavy metals and reactive oxygen species (ROS) (Sun et al., 2002), as well as infection by pathogens (Maimbo et al., 2007; Sarkar et al., 2009; Kandoth et al., 2011).

Recently, extreme global temperatures have become increasingly frequent due to climate change, increasing the frequency and severity of heat damage to crop production. Extreme temperatures can reduce seed vigor, inhibit germination, and restrict plant growth (Yamori et al., 2014). It is important, therefore, to understand the plant heat tolerance. HSP produced under high temperature stress is highly conservative proteins in organisms (Gupta et al., 2010). The abundance of heat shock proteins is typically low under normal conditions, but under heat stress, rapidly grows to account for up to 15% of the total protein content in organisms (Swindell et al., 2007).

Plant HSPs respond to the external environment changes to confer plant thermotolerance by acting as molecular chaperones, maintaining homeostasis of protein folding, and preventing or repairing the misfolding and degradation of proteins (Charng et al., 2006). Plant HSPs can be grouped into five categories according to their molecular weight and sequence homology, including HSP100, HSP90, HSP70, HSP60 and HSP20 (Waters, 2013). HSP20s—also called as small heat shock proteins (sHSPs)—are the most prevalent and abundant proteins in plant HSPs and have a molecular weight ranging from 15 to 42 kDa (Ji et al., 2019). Many HSP20s can form oligomers with high molecular weight and are involved in maintaining the stability of proteins, thus playing a vital role in the formation of plant acquired thermotolerance (Maimbo et al., 2007; Waters, 2013; Haslbeck and Vierling, 2015). For instance, a majority of *HSP20* genes are induced by heat stress in pepper and apple species (Guo et al., 2015; Yao et al., 2020). Additional studies have verified the heat tolerance of HSPs in transgenic plants. The *Populus trichocarpa HSP20* gene *PtHSP17.8* is involved in heat tolerance (Li et al., 2016). The rice HSP20 protein sHsp17.7 confers both heat tolerance and UV-B resistance of transgenic rice plants (Murakami et al., 2004). The transgenic tobacco overexpressing *Zea mays HSP20* gene *ZmHSP16.9* showed increased heat tolerance (Sun et al., 2012). Overall, these studies suggest that *HSP20* genes are key to mediating heat tolerance in plants.

HSP20 bind target proteins through conformational changes to prevent misfolding and irreversible protein aggregation (Hilton et al., 2013). The most prominent feature of HSP20 protein is a highly conserved alpha-crystallin domain (ACD) containing approximately 90 amino acid residues (Waters et al., 1996). This domain is flanked by a variable N-terminal region and a C-terminal extension (Hilton et al., 2013; Waters, 2013). These three regions possess different functions: the ACD participates in substrate interactions, the N-terminus is involved in substrate binding, and the C-terminal extension is responsible for homo-oligomerization (Kirschner et al., 2000; Giese and Vierling, 2004; Basha et al., 2006; Jaya et al., 2009). The ACD domain contained 4 anti-parallel sheets and 3 β-strands, i.e., conserved region I (CRI, β2-β3-β4-β5) in the N-terminus and conserved region II (CRII, β7-β8-β9) in the C-terminus, respectively, separated by a hydrophobic region loop (β6-loop) (Bondino et al., 2012; Haslbeck and Vierling, 2015).

Unlike other HSPs, *HSP20* gene family exhibits extreme sequence variability and evolutionary divergence (Basha et al., 2012). For example, plants contain approximately four times more *HSP20* genes than do animals (Waters et al., 1996). The number of *HSP20* genes in commonly studied or economically important plant species ranges from 19 (*Arabidopsis;* Scharf et al., 2001; Waters, 2013) to 94 (*Gossypium hirsutum*; Ma et al., 2016). Furthermore, plant HSP20s can be divided into different subfamilies based on cellular location, sequence homology or function (Vierling, 1991; Waters et al., 1996). In *Arabidopsis*, HSP20 proteins were divided into 12 subfamilies (Scharf et al., 2001; Waters, 2013), while 15 subfamilies in soybean (LoPes-Caitar et al., 2013), and 14 subfamilies in *G. hirsutum* (Ma et al., 2016).

Pumpkin (*Cucurbita moschata*) is a globally important cucurbit vegetable crop. The top producer of pumpkin is China with 7.7 million tons, followed by India (5 million tons) and Russia (1.2 million tons) (Worldmapper, 2021). Pumpkin exhibits strong resistance to abiotic stress and is often used as rootstock to improve stress tolerance of other cucurbits (Cao et al., 2017). However, the molecular regulatory mechanism underlying pumpkin responses to abiotic stresses are not yet fully understood. In recent years, increasingly frequent temperature extremes have seriously limited the quality and yield of cucurbits. Therefore, it is very important to study the heat resistance mechanism of pumpkin and select strong heat resistant pumpkin rootstock varieties to improve the heat resistance of cucurbits. Study of HSP20s is important for understanding the mechanism of heat tolerance in pumpkin.

In this study, we used bioinformatics methods to identify *HSP20* genes based on the full genomic sequence of *Cucurbita moschata* (Sun et al., 2017) and investigated their physicochemical properties, phylogenetic relationships, conserved domains, gene structures, *cis*-elements and expression patterns in response to heat stress. This study provides foundational information for new research into the functions of *CmoHSP20* gene family and future screening of candidate heat tolerance genes for the improvement of cucurbit vegetable crops.

## Materials and Methods

### Identification of the *HSP20* Genes in *Cucurbita moschata*

The Hidden Markov Model (HMM) profile of the HSP20 domain (PF00011) was taken from Protein family database (Pfam 34.0; http://pfam.xfam.org/). The *Cucurbita moschata* Genome Database (CuGenDB, http://cucurbitgenomics.org/) was searched for this profile using BlastP methods with a cut-off E-value <10^−5^ (Yu et al., 2016). The Pfam, Simple Modular Architecture Research Tool (SMART, v9; http://smart.embl.de/smart/batch.pl) and Conserved Domain Database (CDD, v3.19; https://www.ncbi.nlm.nih.gov/Structure/bwrpsb/bwrpsb.cgi) confirmed the locations of the conserved HSP20 domain. After excluding the redundant sequence lacking the common HSP20 domain or with a molecular weight outside the range of 15–42 kDa (Ji et al., 2019), the final candidate HSP20 proteins of *Cucurbita moschata* were identified. Using the same method, the putative *HSP20* members from *Arabiodpsis thaliana* and *Oryza sativa* were obtained from the TAIR database (https://www.arabidopsis.org/index.jsp) and the Rice Genome Annotation Project (http://rice.plantbiology.msu.edu/), respectively.

### Sequence Analysis, Structural Characterization and Chromosomal Localization

The coding sequences, genomic sequences, and amino acid sequences of the *CmoHSP20* genes were obtained from the CuGenDB. The theoretical isoelectric point (pI) and molecular weight (MW) of each HSP20 protein was estimated using the pI/MW tool from ExPASy (v.3.0; http://web.expasy.org/protparam/), and the number of transmembrane regions per protein was calculated using TMHMM software (v.2.0; http://www.cbs.dtu.dk/services/TMHMM/). The structures of the *HSP20* genes were visualized using the Gene Structure Display Server (GSDS 2.0; http://gsds.cbi.pku.edu.cn). The Multiple Em for Motif Elicitation (MEME) program (MEME Suite 5.3.3; http://meme-suite.org/tools/meme) was used to identify conserved motifs of HSP20 proteins, specifically recording the number of repetitions (any, maximum number of motifs-10, and the optimum motif widths set from 6 to 200 amino acid residues). The results were visualized using TBtools (Chen et al., 2020). The chromosomal position of each *CmoHSP20* gene was acquired from the *C. moschata* genome browser at the CuGenDB and mapped using MapChart software (Voorrips, 2002).

### Phylogenetic Analysis and Classification of *Cucurbita moschata HSP20* Genes

The full amino acid sequences of 97 total HSP20 members from *C. moschata* (*N* = 33), *Arabidopsis* (*N* = 31) and *Oryza sativa* (*N* = 33) were aligned using CLUSTALW (Thompson et al., 1994) program (Supplementary Table S1). MEGA-X (Kumar et al., 2018) was used to construct a phylogenetic tree using the Maximum Likelihood (ML) method with bootstrap test of 1,000 times. The HSP20 proteins were classified into different groups according to the classifications and *in silico* subcellular localization of HSP20 proteins in *O. sativa* and *Arabidopsis* (Waters et al., 1996). The phylogenetic tree was visualized and enhanced using the EvolView online tool (Evolview v3; https://evolgenius.info/evolview-v2).

### Gene Duplication, Collinearity and *Ka/Ks* of *HSP20* Analysis

To investigate the degree of synteny, the homologous regions of the *CmoHSP20* genes were first identified using Multiple Collinearity Scan toolkit (MCScanX) software (Wang et al., 2012). *CmoHSP20* gene duplication events were determined based on whether the length of the shorter gene covered was equal or greater than 70% of the longer gene and if the similarity of the two aligned genes was equal or greater than 70% (Gu et al., 2002; Yang et al., 2008). Tandem and segmental duplications are reported to be the two main mechanisms underlying gene family expansion (Cannon et al., 2004). Genes located on the same chromosome fragment of less than 100 kb and separated by five or fewer genes were considered to be tandem duplicated genes (Wang et al., 2010). Genes found to be coparalogs located on duplicated chromosomal blocks were considered to be segmental duplicated genes (Wei et al., 2007). Tandem and segmental duplication events were visualized using Circos software (v0.69; Krzywinski et al., 2009). Ka/Ks values can be used to predict selection pressure for replicating genes. A Bio-linux system was used to calculate the nonsynonymous (*Ka*) and synonymous (*Ks*) nucleotide substitution parameters. If the ratio of *Ka*/*Ks* was greater than, equal to, or less than one, this indicated positive, neutral, and purifying selection, respectively (Hurst et al., 2002). Syntenic maps were generated using Circos software to display synteny relationships in the orthologous *HSP20* genes obtained from pumpkin and the other focal species.

### Protein 3D Structure and Promoter Analysis of *CmoHSP20* Genes

The tertiary structures of CmoHSP20 proteins were predicted using the online prediction tool SWISS-MODEL (https://swissmodel.expasy.org/) according to the default parameters. The *cis*-acting elements in the 1,500 bp upstream sequences of coding region of *C. moschata HSP20* genes were retrieved from the *C. moschata* genome, and the types, numbers and functions of these elements were analyzed using PlantCARE software (http://bioinformatics.psb.ugent.be/webtools/plantcare/html/).

### Plant Materials and Heat Stress Treatment

Pumpkin cultivar Miben 2 (*Cucurbita moschata* cv. Miben 2) seeds were sprouting in an incubator under 37°C for 2 days under dark condition and then grown in a growth chamber under standard greenhouse conditions (light/dark cycle: 12 h/12 h at 25°C, 70% relative humidity). When seedlings sprouted three true leaves, uniform seedlings were transferred to a high temperature growth chamber (42°C). Root and leaf samples were collected after 0, 3, 6, 12, and 24 h under normal condition (25°C) and heat stress treatment (42°C) and frozen with liquid nitrogen for total RNA extraction and expression analysis. Each treatment was conducted with three independent replicates, and samples from five plants were collected for each replicate.

### Total RNA Extraction and Expression Analyses of *CmoHSP20* Genes

Total RNA was extracted from each sample using an RNAprep Pure Plant kit (Tiangen Biotech (Beijing) Co., Ltd., China, DP441) according to the manufacturer’s instructions. First strand cDNA was synthesized from 1 μg of RNA using the PrimeScript RT reagent kit (TaKaRa, Japan, RR047A). Quantitative RT-PCR (qRT-PCR) was performed to study the expression profiles of *CmoHSP20s* using gene-specific primers from TransStart Tip Green qPCR SuperMix (TransGen Biotech, AQ141-04) with an Applied Biosystems QuantStudio 3 & 5 system (ThermoFisher Scientific, United States). The housekeeping gene *Actin* was used as an internal control. All primers were designed to avoid the conserved region for specificity (Supplementary Table S2).

### Statistical Analysis

The relative expression levels of *CmoHSP20* genes in roots and leaves were calculated by the 2^−△△CT^ method (Livak and Schmittgen, 2001). All data were calculated using the expression level under heat stress divided by that under normal condition at the same time points and presented as the means ± standard error (SE) of three replicates and differences were detected using the Student’s t-test. Asterisk (∗ or ∗∗) indicate a significant difference at *p* < 0.05 or 0.01, respectively.

## Results

### Identification of the HSP20 Proteins in *Cucurbita moschata*

Fourty-three HSP20 proteins were initially obtained by HMM search from pumpkin genome database. After removing the repetitive and incomplete sequences and the sequences with a molecular weight outside of the 15–42 kDa range, 33 sequences were confirmed as *HSP20* genes and named based on their chromosomal locations. Sequences including genomic sequence, transcript sequence, CDS sequence and protein sequence of the gene family were shown in Supplementary Tables S3–S6. For information including gene name, gene identity, chromosomal location, open reading frame (ORF) length, amino acid (AA) number, molecular weight (MW) and isoelectric point (pI) of each *CmoHSP20* gene, see Table 1. *CmoHSP20* genes were distributed on 13 pumpkin chromosomes. The AA number of the CmoHSP20 proteins ranged from 121 (CmoHSP20-31) to 353 (CmoHSP20-21), with an average of 189 AAs. The MW of CmoHSP20s was between 14.29 kDa (CmoHSP20-31) and 39.57 kDa (CmoHSP20-21), with an average of 21.27 kDa, while the pI values of CmoHSP20 ranged from 4.42 (CmoHSP20-1) to 10.08 (CmoHSP20-20), with an average of 7.69. All but six proteins lacked transmembrane domains, suggesting that most CmoHSP20s were non-membrane protein.

**TABLE 1:** Features of *CmoHSP20* genes in pumpkin.

Gene name	Gene ID	Chr.	Genomic location	ORF	AA	MW	pI	No. of transmembrane	Type
CmoHSP20-1	CmoCh01G006670.1	1	3394161…3394613	453	150	16.65	4.42	0	ER
CmoHSP20-2	CmoCh01G007920.1	1	4173468…4174690	471	156	17.37	9.54	1	CIII
CmoHSP20-3	CmoCh01G009310.1	1	5353002…5353550	549	182	21.00	6.98	1	PX/Po
CmoHSP20-4	CmoCh01G009330.1	1	5377056…5377670	615	204	23.36	9.66	0	PX/Po
CmoHSP20-5	CmoCh01G017970.1	1	13240619…13241032	414	137	15.32	6.79	0	CV
CmoHSP20-6	CmoCh01G017990.1	1	13241940…13242419	480	159	17.77	6.29	0	CV
CmoHSP20-7	CmoCh02G008240.1	2	5035698…5036180	483	160	18.08	6.82	0	CI
CmoHSP20-8	CmoCh02G012130.1	2	7301684…7304736	789	262	29.04	9.87	1	CIII
CmoHSP20-9	CmoCh03G003990.1	3	4821790…4822221	432	143	15.93	7.7	0	CI
CmoHSP20-10	CmoCh04G012110.1	4	6165702…6166181	480	159	17.89	5.89	0	CI
CmoHSP20-11	CmoCh04G012120.1	4	6168330…6168779	450	149	16.95	9.66	0	CI
CmoHSP20-12	CmoCh04G012140.1	4	6169337…6169816	480	159	17.78	5.88	0	CI
CmoHSP20-13	CmoCh04G012160.1	4	6177931…6178374	444	147	16.81	9.06	0	CI
CmoHSP20-14	CmoCh04G012170.1	4	6178926…6181730	810	269	30.43	9.85	0	CI
CmoHSP20-15	CmoCh04G019050.1	4	9714250…9718681	711	236	26.63	9.66	0	P
CmoHSP20-16	CmoCh06G002650.1	6	1347700…1348293	594	197	23.07	8.41	0	Unclassified
CmoHSP20-17	CmoCh06G008000.1	6	4285176…4287336	636	211	23.69	5.92	0	MII
CmoHSP20-18	CmoCh09G003550.1	9	1535666…1536418	654	217	24.60	9.34	0	CV
CmoHSP20-19	CmoCh10G010540.1	10	5590943…5591889	510	169	19.15	9.12	1	CIII
CmoHSP20-20	CmoCh10G010550.1	10	5605564…5606784	834	277	30.14	10.08	1	CIII
CmoHSP20-21	CmoCh11G007950.1	11	3938717…3940523	1062	353	39.57	8.87	1	CIII
CmoHSP20-22	CmoCh13G000910.1	13	541183…541665	483	160	18.38	5.4	0	CI
CmoHSP20-23	CmoCh13G000920.1	13	543065…543553	489	162	18.40	4.86	0	CI
CmoHSP20-24	CmoCh13G007610.1	13	7389761…7390857	414	137	15.37	7.18	0	ER
CmoHSP20-25	CmoCh14G000990.1	14	460488…461081	594	197	23.13	9.17	0	Unclassified
CmoHSP20-26	CmoCh14G012350.1	14	10468467…10468943	477	158	17.23	8.2	0	CV
CmoHSP20-27	CmoCh14G013730.1	14	11333320…11337010	675	224	25.64	6.54	0	P
CmoHSP20-28	CmoCh15G001240.1	15	597875…598357	483	160	18.24	5.44	0	CI
CmoHSP20-29	CmoCh15G007840.1	15	3854071…3856991	426	141	15.84	7.85	0	CIV
CmoHSP20-30	CmoCh15G010620.1	15	6921213…6922114	678	225	25.14	8.89	0	P
CmoHSP20-31	CmoCh16G009150.1	16	5520923…5521723	366	121	14.29	6.77	0	MI
CmoHSP20-32	CmoCh19G005900.1	19	6628407…6629798	606	201	22.59	6.52	0	PX/Po
CmoHSP20-33	CmoCh19G010850.1	19	9329362…9333815	735	244	26.42	7.1	0	ER

### Phylogenetic Analysis of CmoHSP20 Proteins

**FIGURE 1:**
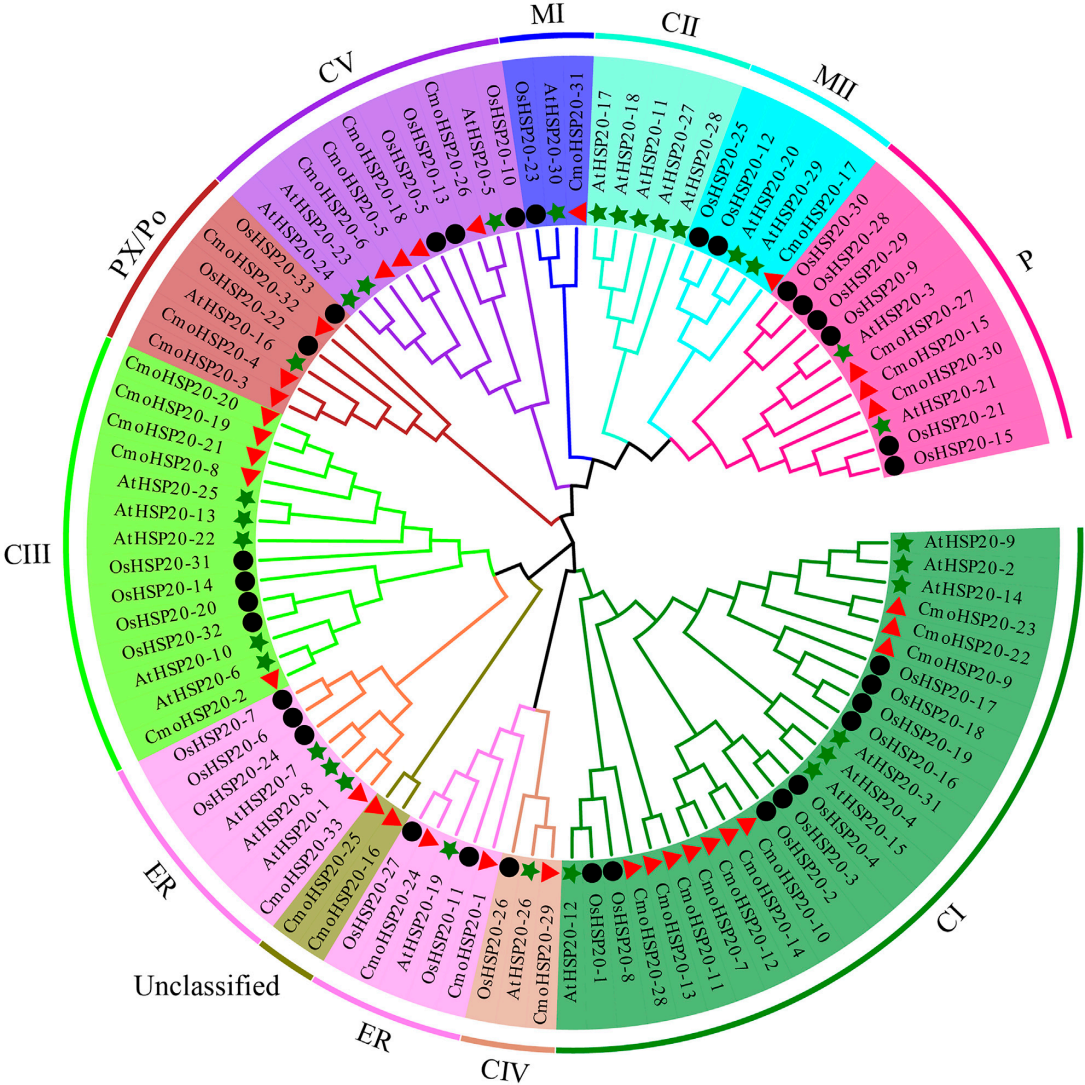
Phylogenetic analyses of HSP20s proteins from *Cucurbita moschata*, *Arabidopsis* and *Oryza sativa*. A phylogenetic tree of HSP2 proteins was constructed using MEGA-X software. The 10 subgroups are indicated with different colors. The red triangles represent *Cucurbita moschata* HSP20s (CmoHSP20s), the green stars represent *Arabidopsis thaliana* HSP20s (AtHSP20s), and the black circles represent *Oryza sativa* HSP20s (OsHSP20s).

To evaluate the evolutionary relationships of pumpkin HSP20 proteins, a ML phylogenetic tree was constructed. The pumpkin HSP20 proteins were divided into nine subfamilies: cytosol I (CI), CIII, CIV, CV, mitochondria I (MI), MII, endoplasmic reticulum (ER), plastid (P), and peroxisome (PX/Po). Cytoplasmic subfamilies (CI to CV) contained 20 members and constituted the largest clade, while no CmoHSP20s were found in subfamily CII. The M (MI and MII) contained two gene members while the ER, P, and PX/Po subfamilies each contained three HSP20s. Two CmoHSP20s (CmoHSP20-16 and CmoHSP20-25) could not be clustered into any subfamily and remained unclassified (Figure 1; Table 1).

### Conserved Motifs and Gene Structures of CmoHSP20s

**FIGURE 2:**
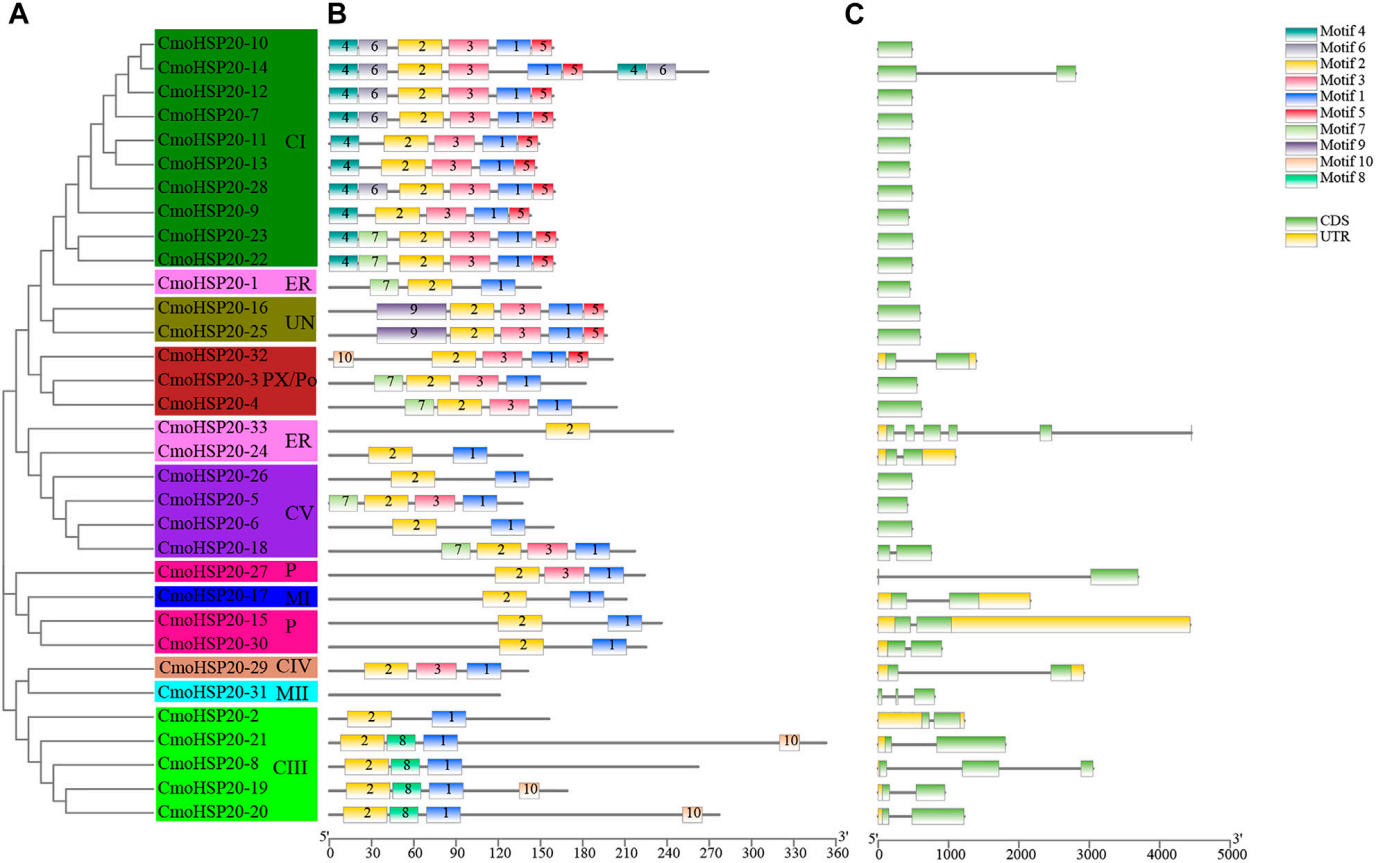
Phylogenetic relationships, structures, and motifs of CmoHSP20 family members. **(A)** A phylogenetic tree of 33 CmoHSP20 proteins constructed using Maximum Likelihood methods. The different subgroups are indicated with different background colors and letters. **(B)** Conserved motifs of CmoHSP20 proteins. Different motifs are represented by colored boxes and different numbers. **(C)** Exon/intron structures of *CmoHSP20* genes. Exons, introns, and UTRs are represented by green boxes, black lines, and yellow boxes, respectively. The phylogenetic tree, conserved motifs, and gene structures were predicted with TBtools.

**FIGURE 3:**
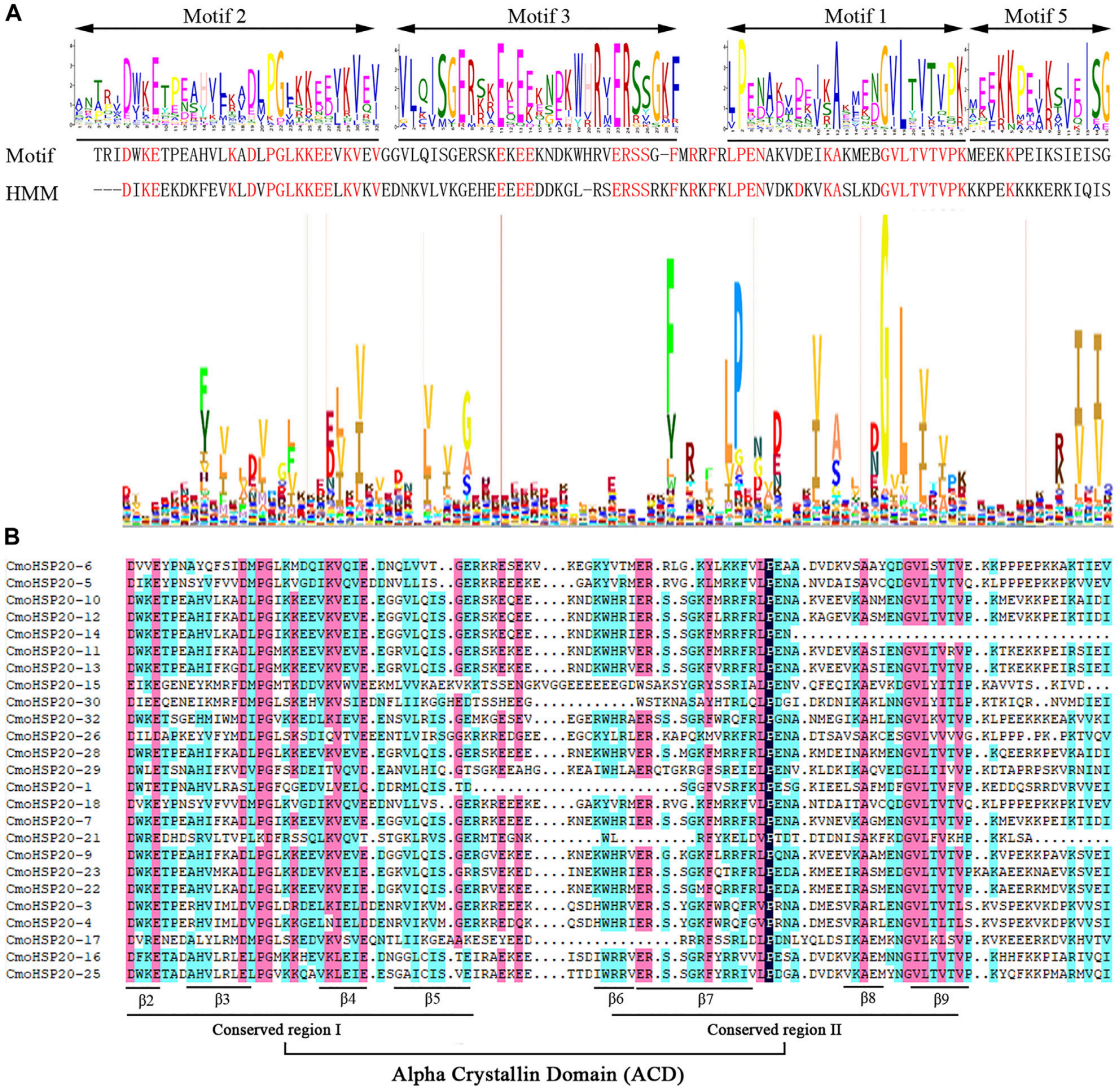
Alignments the ACDs of CmoHSP20s. **(A)** Alignment of the ACD from the MEME results for the CmoHSP20. The motif 2, 3, 1, and 5 formed the putative CmoHSP20 ACD, and the HMM logo from Pfam representing the HSP20 domain (PF00011). The red amino acids represent matches between the MEME motifs and HMM sequences. **(B)** Alignment of the ACDs of CmoHSP20s from *Cucurbita moschata*. Names of all gene members are shown on the left side of the figure. The primary structure of the ACD, including the conserved regions I (CRI), II (CRII), and β6-loop, is shown at the bottom of the figure.

To investigate the structural features of the HSP20 proteins, the conserved motifs were analyzed using MEME. A total of 10 distinct motifs, named motif 1 to motif 10, were detected (Supplementary Figure S1). The lengths of these conserved motifs varied from 8 (motif 8) to 49 (motif 7) AAs. The number of the conserved motifs for each HSP20 protein ranged from 1 to 9. While most CmoHSP20s had 3 to 7 conserved motifs, CmoHSP20-31 and CmoHSP20-33 contained only one motif each (motifs 1 and 2, respectively; Figure 2B). The ACD was formed from two conserved regions, CRI (β2, β3, β4, and β5) and CRII (β7, β8, and β9), which were separated by a hydrophilic domain β6-loop. Based on the Pfam and SMART analyses, the highly conserved ACD is formed from the full sequences of motif 2, 3, 1, and 5 (Figure 3A), in which the combined sequence of motif 2 and 3 contained the CRI of ACD, while the combined sequence of motif 1 and 5 contained the CRII of ACD (Figure 3B). These four motifs were detected in majority of the CmoHSP20 proteins (Figure 2B), with only four proteins (CmoHSP20-1, CmoHSP20-17, CmoHSP20-21, CmoHSP20-30) either lacking the β6-loop or containing variable sequences between β5 and β7.

To understand the evolutionary relationships of pumpkin *HSP20* genes, the exon-intron structures were analyzed (Figure 2C). Among the *HSP20s*, 17 (52%) were intronless, 14 (42%) possessed one intron, and one gene (*CmoHSP20-31*) contained two introns and one gene (*CmoHSP20-33*) contained five introns. Gene structure analysis grouped genes with similar exon-intron patterns into the same clusters (Figures 2A,C).

### Homology Modelling of the CmoHSP20 Proteins

**FIGURE 4:**
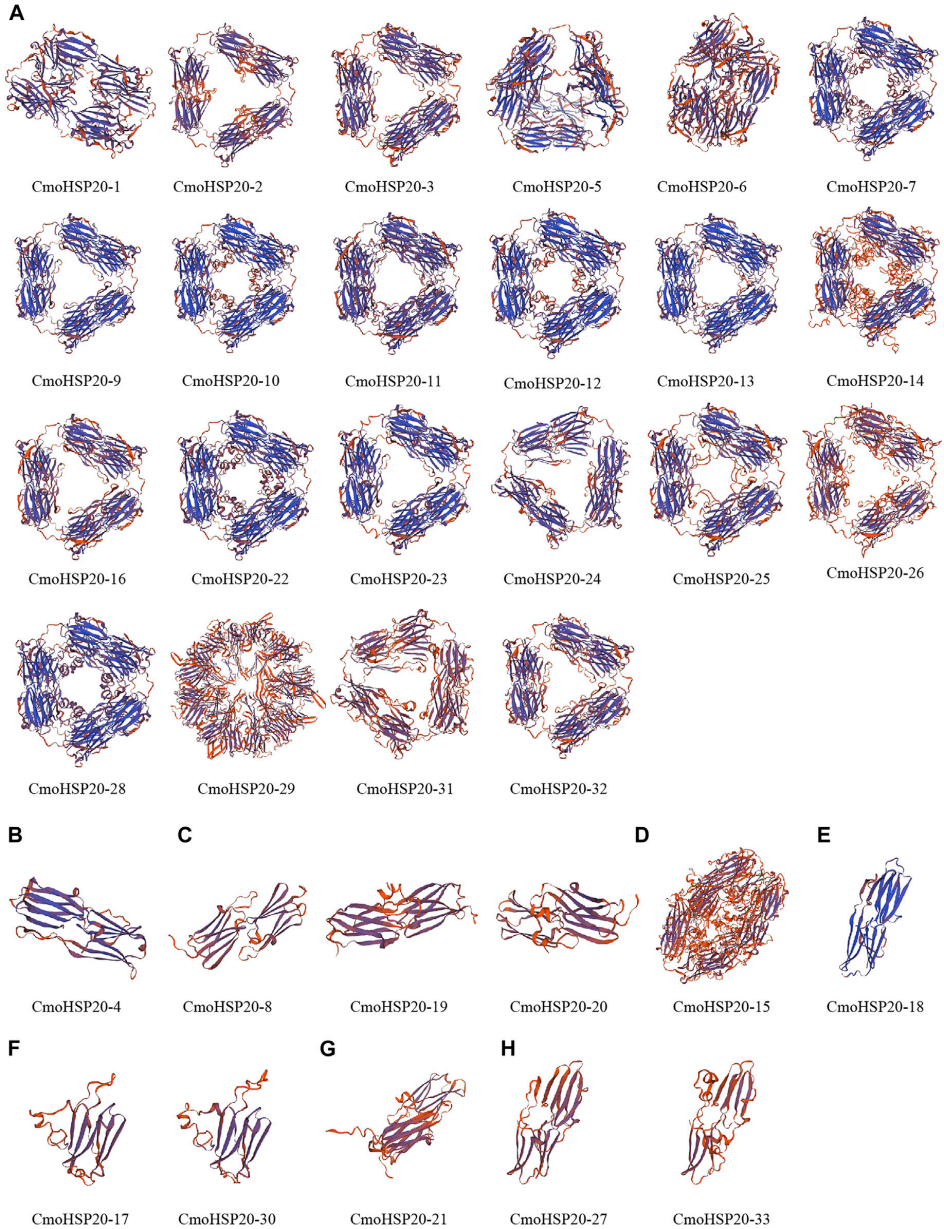
The cartoon representation of the predicted 3-dimensional structural models of CmoHSP20 proteins. The capital letter (A to H) represents the different types.

In order to obtain a reasonable theoretical structure of the CmoHSP20s, protein homology modelling was performed using a SWISS-MODEL server. The predicted 3D structures are shown in Figure 4. Each CmoHSP20 protein was searched for template automatically in the software and then built model using the template (Supplementary Figure S2). The proteins can be divided into eight groups (group A to H) according to their structure similarity. Group A contained 22 proteins, with the most members. Group B, D, E and G all had only one protein. All CmoHSP20 proteins had β-turn, and the protein structures of the same template were basically similar, suggesting that the predicted results were credible.

### Chromosomal Location, Gene Duplication and Synteny Analysis of *CmoHSP20s*

**FIGURE 5:**
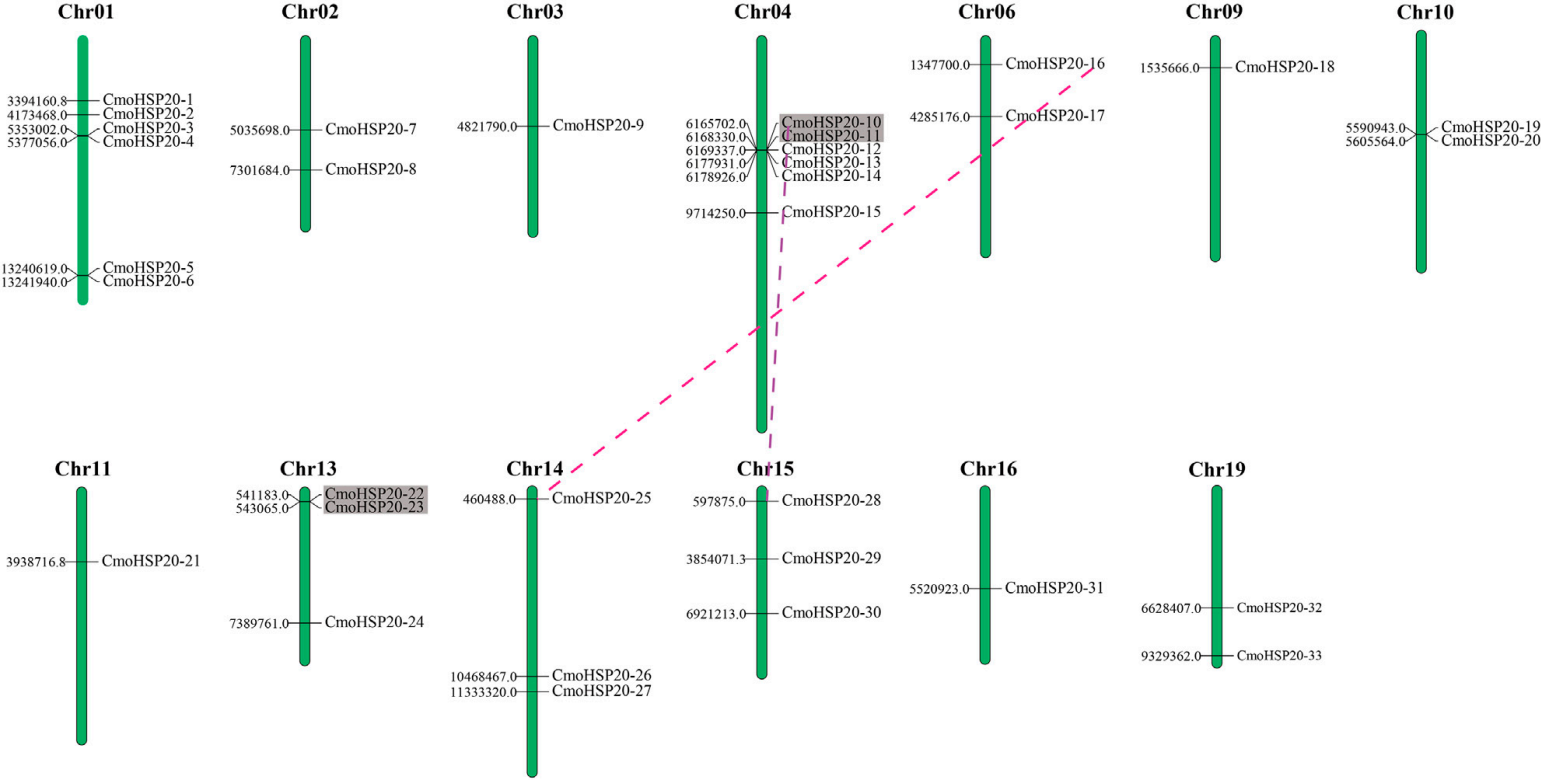
Chromosomal location and gene duplication events in *CmoHSP20s*. The chromosome number is listed at the top of each chromosome while the number to the left of each chromosome represents the location of the *CmoHSP20* gene on the right. Only the chromosomes where *CmoHSP20* genes were mapped are shown. The tandem duplicated genes are marked by grey rectangles and the segmental duplicated genes are linked by colored dotted lines.

A total of 33 *CmoHSP20* genes were mapped on 13 chromosomes (Chr) and exhibited a non-uniform distribution (Figure 5). The maximum number of *CmoHSP20* genes per Chr was six (Chr01 and Chr04) with only a single gene on Chrs 03, 09, 11 and 16*.*

According to the defined criteria, the analysis of gene duplication events showed that there were two pairs of tandem duplication genes (*CmoHSP20-10/-11* and *CmoHSP20-22/-23*) and two pairs of segmentally duplicated genes (*CmoHSP20-10/-28* and *CmoHSP20-16/-25*) in the pumpkin *HSP20* gene family (Figure 5; Supplementary Table S7).

**FIGURE 6:**
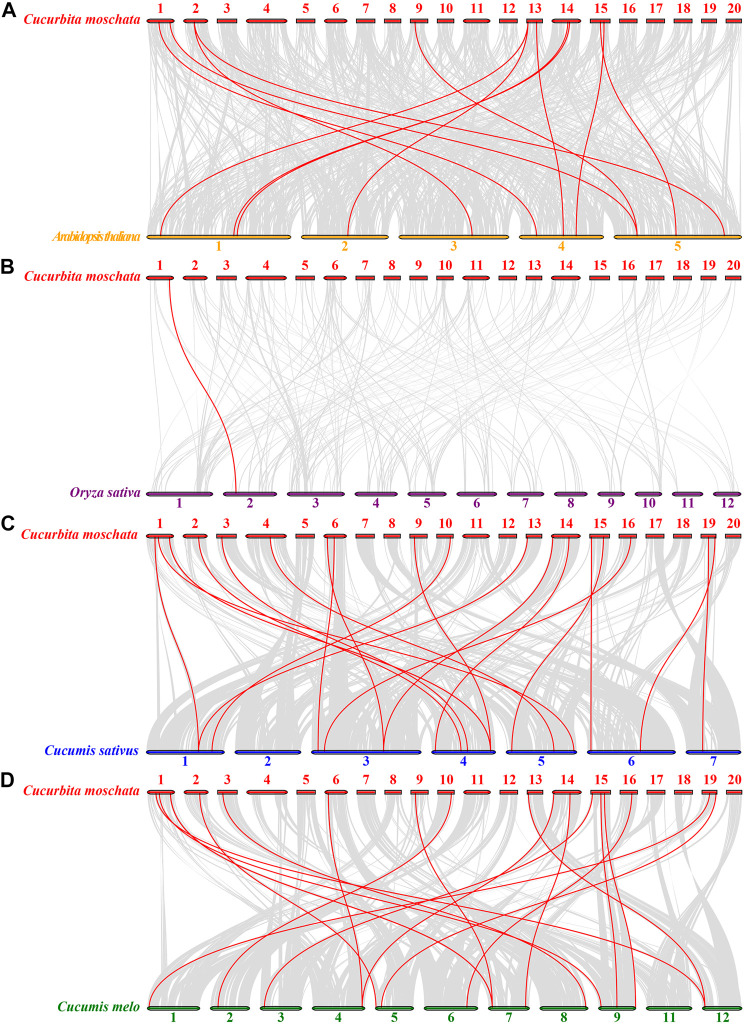
Synteny analyses of *HSP20* genes between *Cucurbita moschata* and four other representative plant species (*Arabidopsis thaliana*, *Oryza sativa*, *Cucumis sativu* and *Cucumis melo*). (**A**) *C. moschata* and *A. thaliana*. (**B**) *C. moschata* and *O. sativa*. (**C**) *C. moschata* and *Cucumis sativu*. (**D**) *C. moschata* and *Cucumis melo*. Gray lines indicate significantly collinear blocks within and among plant genomes, while red lines highlight syntenic *HSP20* gene pairs. The chromosome number is indicated at the top of each chromosome.

To understand the phylogenetic relationships of the *HSP20* genes with other species, comparative synteny maps of four related genomes (*C. moschata* VS *A. thaliana*, *C. moschata* VS *O. sativa*, *C. moschata* VS *Cucumis sativus*, and *C. moschata* VS *Cucumis melo*) were created. Twelve *CmoHSP20* genes displayed a syntenic relationship with genes in *Arabidopsis*, 19 with those in *C. sativus*, 18 with those in *C. melo* and only 1 syntenic relationship with *O. sativa* genes (Figure 6; Supplementary Table S8). The number of collinear gene pairs between pumpkin and other members of Cucurbitaceae (cucumber or melon) was greater than that between more distantly-related *Arabidopsis* or rice, with the number of collinear gene pair being lowest between pumpkin and rice.

The ratio of Ka/Ks for each orthologous *CmoHSP20* gene pair was used to evaluative the type and strength of selective pressure. Both the segmental duplicated *CmoHSP20* gene pairs possessed a *Ka/Ks* ratio <1 (purifying selection), with the higher in the CmoHSP20-16/CmoHSP20-25 pair (*Ka/Ks* value = 0*.*26).

### Promoter Analysis of *CmoHSP20* Genes

**FIGURE 7:**
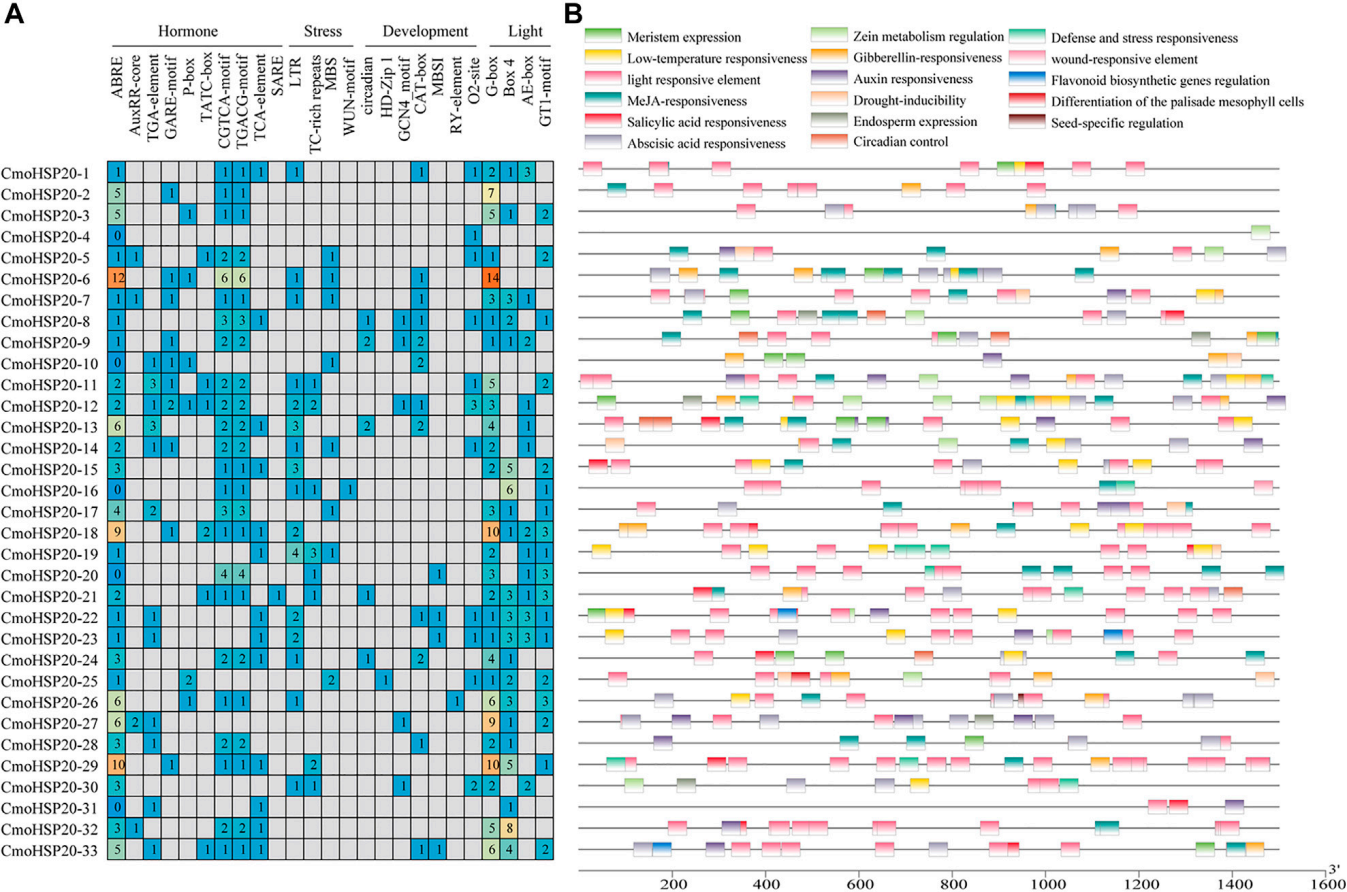
*Cis*-elements analysis of the *CmoHSP20* genes promoter regions. **(A)** The different colors and numbers indicated the numbers of different promoter elements in the *CmoHSP20* genes. **(B)** Colored blocks represent the different types of *cis*-elements and their locations in each *CmoHSP20* gene. The types, numbers, and locations of potential elements in the promoter regions 1.5-kb upstream of the *CmoHSP20* genes were determined using PlantCARE software.

To understand the role of *cis*-regulatory elements in *CmoHSP20*, *cis*-elements were identified in the 1.5 kb upstream sequence from the translation start site (ATG) of each *CmoHSP20* (Figure 7; Supplementary Table S9). Four types of *cis*-elements, including hormone responsive, stress-responsive, plant development-related, and light responsive elements were identified. The largest number of *cis-*elements observed across the 33 *CmoHSP20* genes was associated with light-related responsiveness, such as G-box, Box 4, AE-box and GT1-motif. The hormone-related *cis*-acting elements, including abscisic acid responsiveness (ABRE), auxin responsiveness (AuxRR-core and TGA-element), gibberellin responsive elements (GARE-motif, P-box, and TATC-box), MeJA-responsive (CGTCA-motif and TGACG-motif), and salicylic acid-responsive (TCA-element and SARE), were widely present in the promoter region. Among these elements, ABRE, CGTCA-motif and TGACG-motif accounted for the largest part of the hormone responsive category, while the SARE element was only found in the promoter region of *CmoHSP20-21*. The stress-related category *cis*-elements containing abiotic stress-related elements (LTR, TC-rich repeats and MBS) and biotic stress-related elements (WUN-motif), as well as plant development-related elements, including circadian control (circadian), differentiation of the palisade mesophyll cells (HD-Zip 1), endosperm expression (GCN_4 motif), meristem expression (CAT-box), flavonoid biosynthetic genes regulation (MBSI), seed-specific regulation (RY-element), and zein metabolism regulation (O2-site) were also identified. Only *CmoHSP20-25* promoter region contained HD-Zip 1 element, and only *CmoHSP20-26* promoter region contained RY-element element.

### Expression Patterns of *CmoHSP20* Genes Under Heat Stress

**FIGURE 8:**
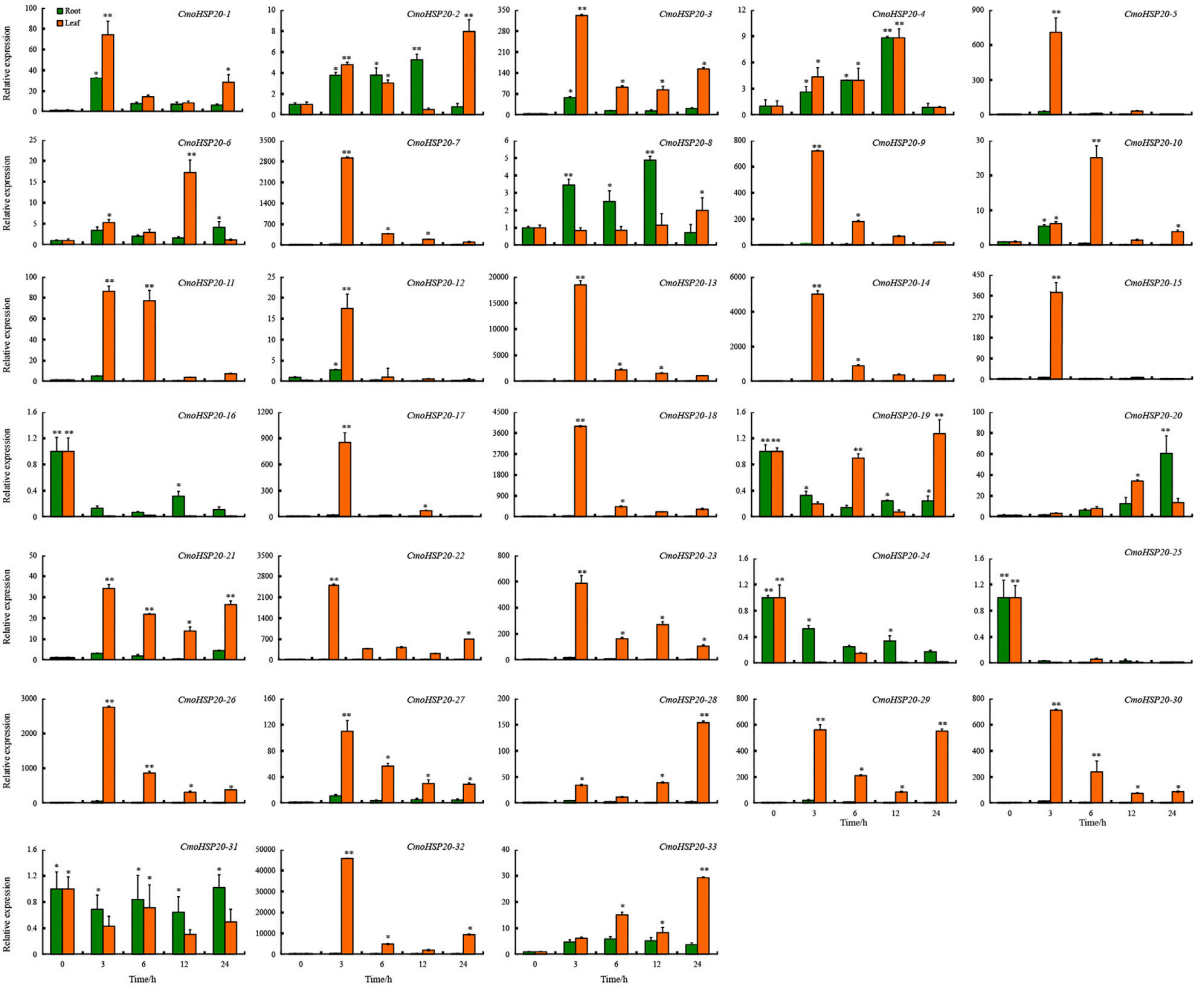
Expression analyses of the *Cucurbita moschata CmoHSP20* genes in response to heat stress using qRT-PCR. The mean expression value was calculated from three replicates. Vertical bars indicate the standard deviation. Values of 0, 3, 6, 12, and 24 indicate hours after treatment. Mean values and standard deviations are calculated according the data. Asterisk (∗ or ∗∗) indicate a significant difference at *p* < 0.05 or 0.01, respectively.

To investigate the expression changes of pumpkin *HSP20* genes in response to heat stress, qRT-PCR was used to examine the transcript levels of the 33 *CmoHSP20* genes. Overall, the relative expression level of *CmoHSP20* genes fluctuated during the 24 h treatments under heat stress conditions (Figure 8). Compared to roots, the expression levels of almost all the genes in leaves were extremely up-regulated. For example, *CmoHSP20-3*, *-5*, *-7*, *-9*, *-13*, *-14*, *-15*, *-17*, *-18*, *-22*, *-23*, *-26*, *-29*, *-30* and *-32* were highly induced in leaves after short-term heat stress (42°C for 3 h), with the expression levels were more than 300-fold than under normal condition. The *CmoHSP20-16*, *-24* and *-25* genes were down-regulated under heat stress in both roots and leaves, while the expression level of *CmoHSP20-31* did not change within neither roots nor leaves in response to heat stress.

## Discussion

Environmental stress is one of the most important environmental and economic challenges in the world today, with the potential to dramatically decrease crop yield and quality across agricultural systems. Small heat shock proteins are widely prevalent across plants species and are rapidly synthesized in response to environmental stress (Wang et al., 2004). Many studies have shown that members of the *HSP20* gene family produce heat shock proteins and are widely involved in abiotic stress in plants (Yu et al., 2016; Zhao et al., 2018). These family members appear to be highly conserved across plant species (Aghdam et al., 2013). With the wide availability of genomic information, the functions of the HSP20 family genes in many plants have now been characterized, including the model plants *Arabidopsis* (Scharf et al., 2001) and rice (Sarkar et al., 2009), as well as soybean (Lopes-Caitar et al., 2013), watermelon (He et al., 2019), potato (Zhao et al., 2018), pepper (Guo et al., 2015), grape (Ji et al., 2019) and apple (Yao et al., 2020). A comprehensive identification and analysis of *HSP20* gene in pumpkin will add important information to this collective knowledge while revealing their functions in pumpkin stress resistance.

With the successful genome sequencing of Cucurbitaceae plants such as pumpkin, it is possible to identify members of the *HSP20* gene family at the whole genome level (Sun et al., 2017). In this study, 33 *HSP20* gene family members were identified from the pumpkin genome database using bioinformatics methods. We found that the number of pumpkin *HSP20* genes was similar to those in *Arabidopsis* (*N* = 31) and *O. sativa* (*N* = 33) (this study), as well as *Capsicum annuum* (*N* = 35) (Guo et al., 2015), but lower than that of *Gossypium hirsutum* (*N* = 94) (Ma et al., 2016). These findings suggest the possibility of a gene gain event during the evolutionary process from diploid (*Arabidopsis*, *O. sativa*, *C. annuum* and *C. moschata*) to tetraploid (*G. hirsutum*).

To determine the evolutionary relationships of *HSP20* genes, we constructed a phylogenetic tree based on the AA sequences of *C. moschata*, *A. thaliana* and *O. sativa* HSP20s. Our phylogenetic analysis indicated that the pumpkin HSP20 family could be divided into nine subfamilies, including 20 HSP20 proteins located in cytoplasm belonged to CI, CIII, CIV and CV, two proteins located in mitochondria belonged to MI and MII, three located in endoplasmic reticulum, three in the plastids, and three in the peroxisome. Two proteins could not be clustered into any subfamily (Guo et al., 2015). These patterns are similar to the distribution characteristics of HSP20 family members in *Arabidopsis* and *O. sativa* (Scharf et al., 2001; Sarkar et al., 2009), indicating a close relationship among HSP20s from pumpkin, *Arabidopsis* and *O. sativa*. This suggests that the biological function of pumpkin HSP20s may be predicted based on the activity of similar genes in these other species. In addition, over 50% of CmoHSP20s were classified into CI-CV subfamilies, providing evidence that the cytoplasm could be the primary functional area of the HSP20 family in pumpkin. However, no HSP20 members belonged to CII family, a finding inconsistent with that from the other focal plants (Lopes-Caitar et al., 2013; Guo et al., 2015; Yao et al., 2020). Possibly, the CII subfamily appeared before pumpkin speciation through multiple gene duplication. Unexpectedly, pumpkin CmoHSP20 members were more closely related to those in the same subfamily from different species than to the other HSP20s from the same species, implying a relatively high synteny between the same HSP20 subfamily across plant species.

The exon/intron structure plays an important role in the evolution of multiple gene families (Xu et al., 2012). Approximately 94% of the *CmoHSP20* genes (predominantly belonging to the CI and CV subfamilies) have lack or possess only a single intron (Figure 2). This result is not unexpected, as plants overall tend to retain genes with no intron or less intron (Mattick and Gagen, 2001). *HSP20* gene families are one of the rapidly expressed genes under environmental stresses (Sarkar et al., 2009; Yu et al., 2016). Here, we tested the expression patterns of pumpkin *HSP20* genes under heat stress and found that most *HSP20* genes were up-regulated after heat treatment.

Presence of conserved motif was also investigated to further study the evolution of pumpkin HSP20 proteins. We found that 67% of the CmoHSP20 proteins had three to seven conserved motifs and almost all the proteins contained motif 2 and motif 1, consisting of the ACD. Furthermore, we found that most HSP20s in the same subfamilies showed conserved motifs and similar gene structures, a finding also reported in tomato and apple (Yu et al., 2016; Yao et al., 2020). This phenomenon supported their close evolutionary relationship and classification of the subfamilies. The features of conserved motifs and gene structures of *CmoHSP20* family may facilitate the identification of additional functions of *CmoHSP20* genes, such as in responses to different types of stressors.

Gene duplication events play a major role in genomic rearrangements and expansions (Vision et al., 2000), allowing an increase in functional divergences that enables plants to adapt to changing environmental conditions (Conant and Wolfe, 2008; Grassi et al., 2008; Flagel and Wendel, 2009). The 33 *CmoHSP20* genes were unevenly dispersed on 13 chromosomes of pumpkin, and five clusters with at least two *CmoHSP20* genes each were identified (Figure 5). A number of family members gathered into clusters in certain segments, especially in Chrs 04 and 13. We discovered two pairs of predicted tandem duplicated genes and two pairs of predicted segmental duplicated genes, suggesting that the two duplicated events contributed to the expansion of *CmoHSP20* family. Furthermore, both the segmental duplicated genes were found to have undergone strong purifying selective pressure, confirming that the evolutionary pattern of *CmoHSP20* genes was highly conservative.

In addition, numerous hormone responsive, stress-responsive, plant development-related and light responsive elements were found in the promoter regions of *CmoHSP20* genes (Figure 7; Supplementary Table S9), indicating that pumpkin *HSP20* genes performed multiple or specific functions. All *CmoHSP20* genes contained light responsive *cis*-elements, suggesting that the pumpkin *HSP20s* were essential in plant growth and development. It has been demonstrated that HSP20s played key roles in the control of plants’ response to environmental stress (Neta-Sharir et al., 2005; Guo et al., 2015; Zhao et al., 2018; He et al., 2019; Ji et al., 2019). We found that most of these genes were up-regulated under heat stress. We also tested the expression levels of other HSP and heat shock transcription factors, and found that the *CmoHsfA2*, *CmoHSP70* and *CmoHSP83* were all induced by heat stress, and showed similar expression patterns with *CmoHSP20* genes (Supplementary Figure S3). It is worth noting that the relative expression levels of 6 *HSP20* genes (*CmoHSP20-7*, *13*, *18*, *22*, *26* and *32*) were extremely up-regulated after 3 h of heat stress. These genes might be mainly involved in the heat stress biological pathway and could be used as candidate genes for heat resistant breeding of *Cucurbitaceae* vegetable crops.

## Conclusion

In this study, a genome-wide identification of *HSP20* genes in *C. moschata* was performed and a total of 33 *CmoHSP20* genes were identified. These genes are unequally distributed on 13 chromosomes and were classified into nine subfamilies based on their phylogenetic relationships. The basic features, genome distribution, gene structures, conserved motifs, gene duplication events, and *cis*-elements of these genes were analyzed, providing a foundational understanding of the evolutionary relationships within the *HSP20* gene family. *CmoHSP20* genes expression was studied using qRT-PCR, results from which revealed that 6 pumpkin *HSP20* genes were highly induced by heat stress. Our results provide a basis for identifying important candidate *HSP20* genes involved in pumpkin responses to heat stress.

## Data Availability

The original contributions presented in the study are included in the article/Supplementary Material, further inquiries can be directed to the corresponding authors.
