# Supplementary figures and images for: Pumpkin (Cucurbita moschata) HSP20 Gene Family Identification and Expression Under Heat Stress
Source: Front Genet. 2021 Oct 14;12:753953. doi: 10.3389/fgene.2021.753953 (PMC8553033; doi:10.3389/fgene.2021.753953)

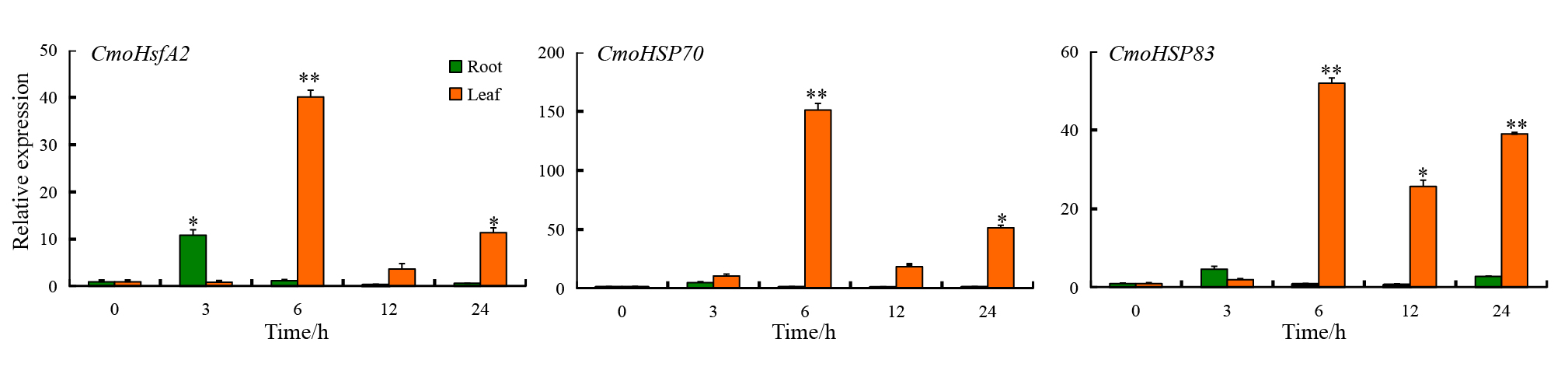

Supplement: Supplementary file 2 [file Image3.JPEG]

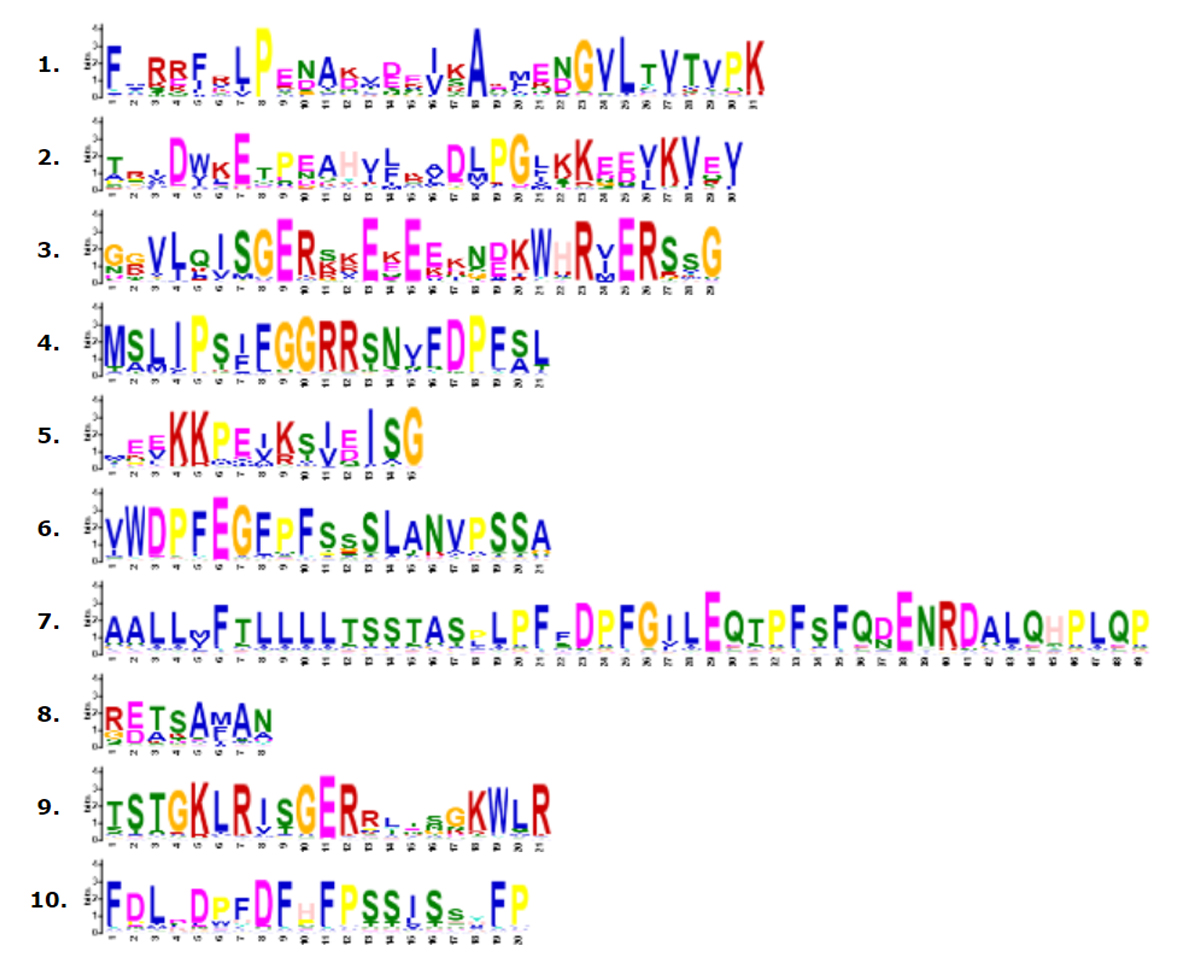

Supplement: Supplementary file 5 [file Image1.JPEG]

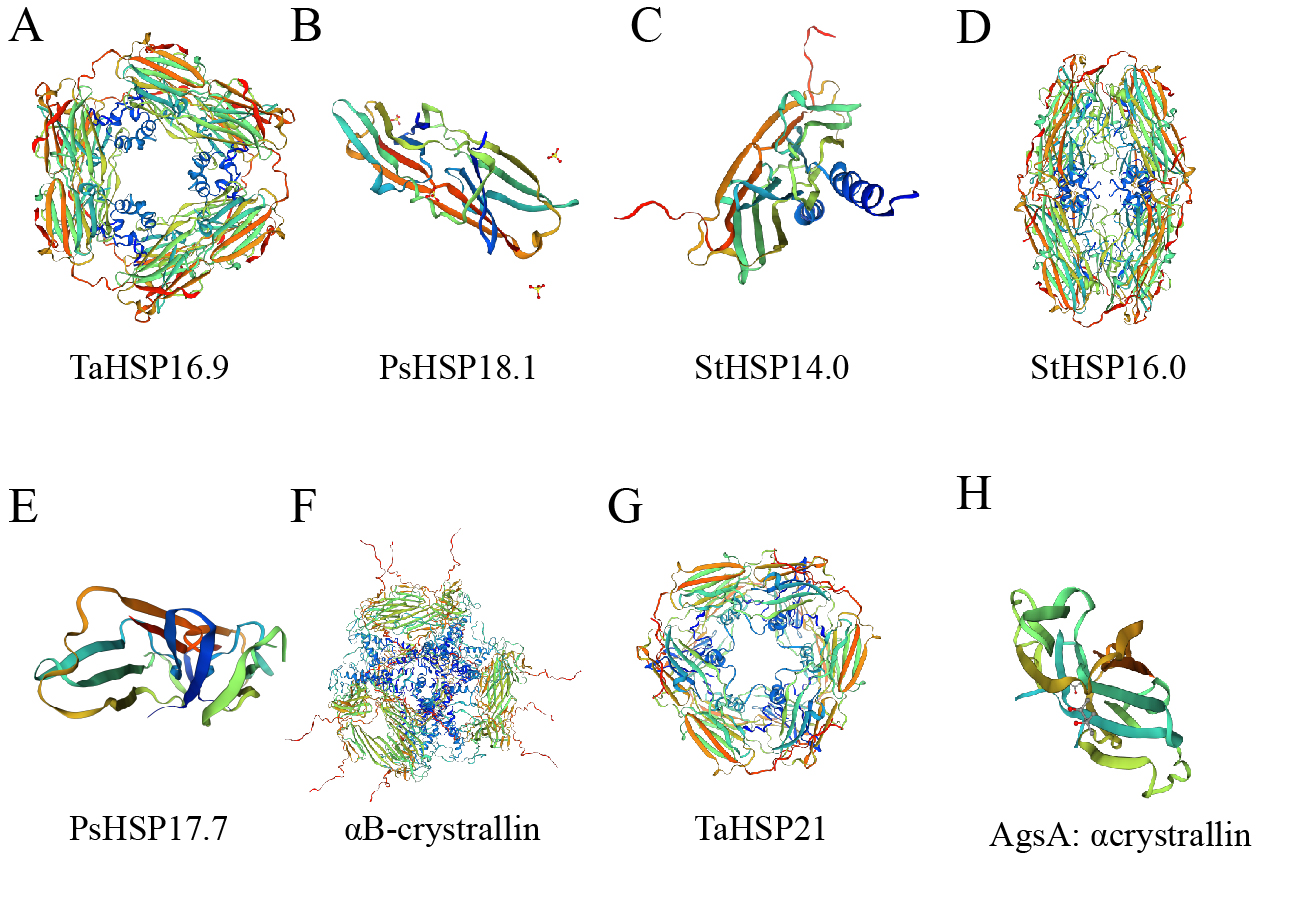

Supplement: Supplementary file 7 [file Image2.JPEG]
