# Supplementary material for: Pumpkin (Cucurbita moschata) HSP20 Gene Family Identification and Expression Under Heat Stress
Source: Front Genet. 2021 Oct 14;12:753953. doi: 10.3389/fgene.2021.753953 (PMC8553033; doi:10.3389/fgene.2021.753953)
Supplement: Supplementary file 12 [file DataSheet1.docx]

Supplementary Material

**Supplementary Figure S1** The amino acid sequences of each motif identified in CmoHSP20 proteins.

**Supplementary Figure S2** The cartoon representation of the predicted 3-dimensional structural models of the template proteins.

**Supplementary Figure S3** Expression analyses of the *Cucurbita moschata* *CmoHsfA2*, *CmoHSP70* and *CmoHSP83* in response to heat stress using qRT-PCR.

**Supplementary Table S1** The gene ID of *HSP20* genes in *C. moschata*, *A. thaliana* and *O. sativa*.

**Supplementary Table S2** Primers used in this study.

**Supplementary Table S3** Genomic sequences of CmoHSP20 gene family.

**Supplementary** **Table S4** mRNA sequences of CmoHSP20 gene family.

**Supplementary** **Table S5** CDS sequences of CmoHSP20 gene family.

**Supplementary** **Table S6** Protein sequences of CmoHSP20 gene family.

**Supplementary** **Table S7** Sequence similarity of duplicated genes.

**Supplementary Table S8** All gene pairs of four different genomes (*C. moschata* VS *A. thaliana*, *C. moschata* VS *O. sativa*, *C. moschata* VS *Cucumis sativus*, and *C. moschata* VS *Cucumis melo*).

**Supplementary Table S9** Information on cis-elements of *CmoHSP20* gene promoters.
